# Multi-Target Inhibitor CUDC-101 Impairs DNA Damage Repair and Enhances Radiation Response in Triple-Negative Breast Cell Line

**DOI:** 10.3390/ph17111467

**Published:** 2024-11-01

**Authors:** Elsie Neo Seane, Shankari Nair, Charlot Vandevoorde, Alessandra Bisio, Anna Joubert

**Affiliations:** 1 Department of Radiography, School of Health Care Sciences, Faculty of Health Sciences, University of Pretoria, Pretoria 0028, South Africa; 2 Department of Medical Imaging and Therapeutic Sciences, Faculty of Health and Wellness, Cape Peninsula University of Technology, Bellville 7535, South Africa; 3 Separate Sector Cyclotron (SSC) Laboratory, Radiation Biophysics Division, iThemba LABS, Cape Town 7530, South Africa; s.nair@ilabs.nrf.ac.za; 4 Department of Biophysics, GSI Helmholtzzentrum für Schwerionenforschung, 64291 Darmstadt, Germany; c.vandevoorde@gsi.de; 5 Department of Cellular, Computational and Integrative Biology, Via Sommarive, 9, Povo, 38123 Trento, Italy; alessandra.bisio@unitn.it; 6 Department of Physiology, School of Medicine, Faculty of Health Sciences, University of Pretoria, Pretoria 0028, South Africa; annie.joubert@up.ac.za

**Keywords:** Histone deacetylase inhibitors, CUDC-101, proton therapy, proton irradiation

## Abstract

Background: Since the discovery that Histone deacetylase inhibitors (HDCAi) could enhance radiation response, a number of HDACi, mainly pan-HDAC inhibitors, have been studied either as monotherapy or in combination with X-ray irradiation or chemotherapeutic drugs in the management of breast cancer. However, studies on the combination of HDACi and proton radiation remain limited. CUDC-101 is a multitarget inhibitor of Histone deacetylases (HDACs), epidermal growth factor receptor (EGFR), and human epidermal growth factor receptor 2 (HER-2). In this paper, the effectiveness of CUDC-101 in enhancing radiation response to both proton and X-ray irradiation was studied. Methods: MCF-7, MDA-MB-231, and MCF-10A cell lines were pre-treated with CUDC-101 and exposed to 148 MeV protons, and X-rays were used as reference radiation. Colony survival, γ-H2AX foci, apoptosis, and cell cycle analysis assays were performed. Results: γ-H2AX foci assays showed increased sensitivity to CUDC-101 in the MDA-MB-231 cell line compared to the MCF-7 cell line. In both cell lines, induction of apoptosis was enhanced in CUDC-101 pre-treated cells compared to radiation (protons or X-rays) alone. Increased apoptosis was also noted in CUDC-101 pre-treated cells in the MCF-10A cell line. Cell cycle analysis showed increased G2/M arrest by CUDC-101 mono-treatment as well as combination of CUDC-101 and X-ray irradiation in the MDA-MB-231 cell line. Conclusions: CUDC-101 effectively enhances response to both proton and X-ray irradiation, in the triple-negative MDA-MB-231 cell line. This enhancement was most notable when CUDC-101 was combined with proton irradiation. This study highlights that CUDC-101 holds potential in the management of triple-negative breast cancer as monotherapy or in combination with protons or X-ray irradiation.

## 1. Introduction

Megavoltage (MV) X-ray-based radiation therapy is commonly indicated as adjuvant therapy to reduce the risk of loco-regional recurrence of breast cancer and to improve disease-free survival [1]. Recent advances in treatment planning and delivery techniques such as intensity modulated radiotherapy (IMRT) and volumetric modulated arc therapy (V-MAT) have improved dose distribution and sparing of healthy tissues; however, associated late side-effects such as secondary malignancies and cardiopulmonary toxicities are still observed in breast cancer survivors [2,3,4,5]. While X-ray-based therapy is widely available, the number of proton therapy centres across the globe is on the rise, and this has improved access for breast cancer patients. In general, the number of cancer patients treated with proton therapy is increasing rapidly with an estimated 190,000 in 2018, and an expected increase to over 300,000 in 2030 [6]. Furthermore, the superior dose distribution of protons makes it also a suitable modality for re-irradiation in a number of tumour types [7]. The physical aspects of proton therapy are well understood, but the biological aspects remain under-explored for protons alone as well as in combination therapies with drugs and concomitant therapies [5,8,9]. In recent years, combination therapies of Histone deacetylase inhibitors (HDACi) and photon irradiation have been a focus of many studies [10,11,12,13,14,15,16]. However, combination therapies with particle type of radiation and HDCAi remain limited.

Histone deacetylase inhibitors (HDACi) are epigenetic drugs that can sensitise cancer cells to ionising radiation with little effect on healthy cells [17,18]. To date, five HDACi SAHA (generic name vorinostat), belinostat, panabinostat, chidamide, and romidepsin have been approved by the Food and Drug Administration (FDA) for cancer therapy [19,20]. More than 20 different HDACi are in different phases of clinical trials as monotherapy or in combination with other DNA damaging agents [21]. HDACi are classified into several classes according to their chemical structure: benzamides (e.g., chidamide, entinostat), hydroxamic acids (e.g., SAHA, belinostat, panabinostat, CUDC-101), cyclic tetrapeptides (e.g., romidepsin), and aliphatic acids (e.g., butyrate, valproic acid) [16,22]. Of these classes, hydroxamic acids is the main class that has been used and is continuously being applied in most studies [23]. This class is also preferred as they inhibit a broad range of HDACs (HDACs1–11), and they have been shown to cause cellular effects at low (nM) concentrations [21].

Evidence from pre-clinical studies has revealed that the combination of photon radiation and HDACi results in increased cell death in a number of cell lines including lung, melanoma, prostate, glioma, colon, non-small cell lung cancer (NSCLC), osteosarcoma, and breast to name a few [10,11,12,13,14,15,16,24,25,26,27]. Reports on combination of HDACi with proton irradiation and particle radiation in general remain limited [28,29,30,31,32]. For treatment of breast cancer, several studies have explored the use of pan-HDACi SAHA or Panobinostat in combination with chemotherapeutic drugs, but studies on CUDC-101 are lacking [33,34,35,36]. CUDC-101 is a hydroxamic acid that inhibits HDACs, epidermal growth factor receptor (EGFR), and human epidermal growth factor receptor 2 (HER-2) [37]. EGFR and HER-2 have been recognized as biomarkers for resistance in tumours. EGFR is reported to be expressed in all molecular sub-types of breast cancer and over-expressed in triple-negative breast cancers [38,39]. Therefore, the dual targeting of EGFR and HER-2 may be beneficial for EGFR over-expressing triple-negative breast cancer [40], for which CUDC-1010 would be an appealing HDACi candidate. To the best of our knowledge, only one in vitro study has been conducted on the effect of CUDC-101 and X-ray irradiation in the triple-negative breast cancer cell line MDA-MB-231 [14]. Considering the growing interest in using proton therapy for breast cancer patients, we aimed to quantify and compare the efficacy of CUDC-101 in combination proton and X-ray radiation, in MCF-7, MCF-10A, and MDA-MB-231 cell lines. The results of this in vitro study serve to highlight the potential of CUDC101 in the treatment of breast cancer, to motivate further in vivo preclinical work and to guide future clinical trials.

## 2. Results

### 2.1. Determination of IC_50_ and Timepoint of Irradiation in Relation to the Drug

To determine the half maximal inhibitory concentration (IC_50_) of HDACi, MCF-7, MDA-MB-231, and MCF-10A cells were pre-treated with CUDC-101 at concentrations that ranged from 0.16 µM to 20 µM. Cell proliferation was assesed 72 h post-treatment using 3-(4,5-dimethylthiazol-2-yl)-2,5-diphenyl-2H-tetrazolium bromide (MTT) cell viability assays. The determined half-maximal inhibitory concentration (IC_50_) values are presented in Table 1.

**Figure 1:**
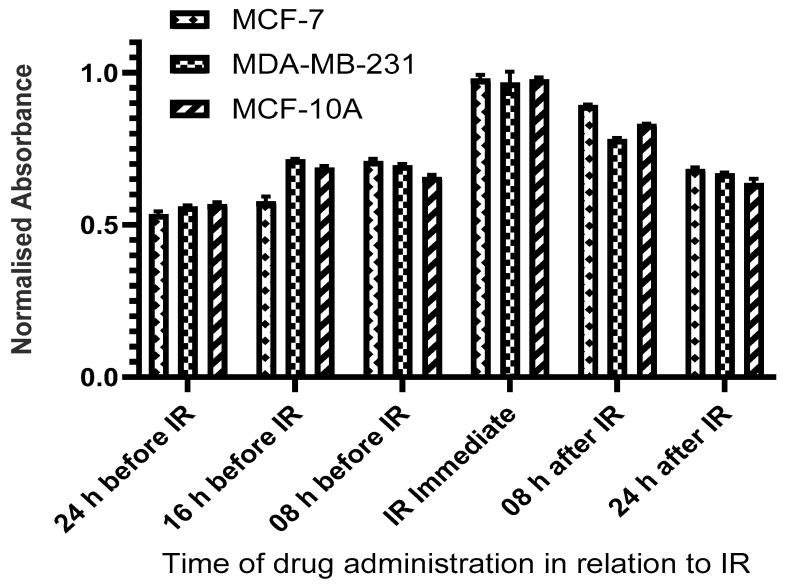
Pre-treatment with CUDC-101 at 24 h before 250 kVp X-ray irradiation offered maximal sensitization. Cells were pre-treated with 0.3 µM, 0.6 µM, and 2.7 µM CUDC-101 in MCF-7, MDA-MB-231, and MCF-10A cell lines. Cell proliferation was evaluated at 24, 16, 8 h before irradiation, immediately, and at 8 and 24 h after irradiation, as depicted on the *x*-axis. Cell proliferation was assessed with MTT assay at 72 h post irradiation.

To determine the optimal sequence of administration of HDACi and radiation, cells were irradiated with X-rays (2 Gy) at 8 h, 16 h, and 24 h before treatment with CUDC-101 as well as immediately, at 8 h, 16 h, and 24 h post-treatment with CUDC-101. Cell proliferation was determined at 72 h post-irradiation using MTT assays. Pre-treatment with HDACi 24 h before irradiation resulted in the least cell survival for all three cell lines and was subsequently used for all experiments (Figure 1).

### 2.2. Effect of CUDC-101 in Enhancing Radiation Response in Breast Cell Lines

**Figure 2:**
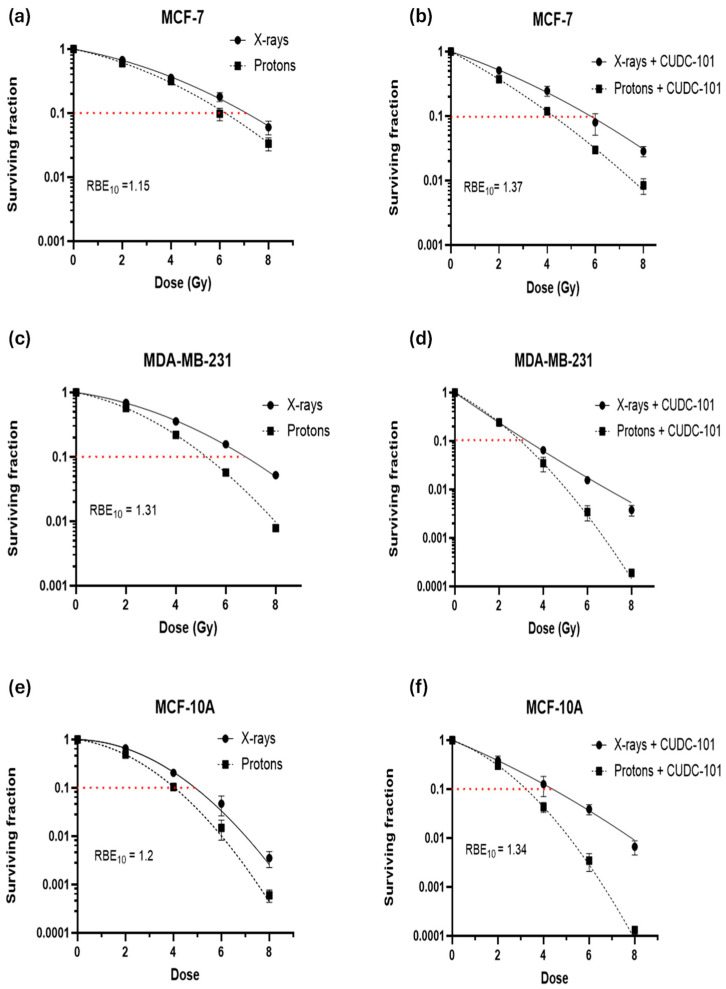
Colony survival curves and associated RBE calculations. CUDC-101 sensitises MCF-7 (**a**,**b**), MDA-MB-231 (**c**,**d**), and MCF-10A (**e**,**f**) cells to proton and X-ray irradiation. Data are expressed as the mean ± SD of three independent experiments.

Colony survival assays were performed to determine the effect of CUDC-101 in enhancing radiation-mediated cell killing (or inhibition of cell proliferation). For all three cell lines, comparison of survival curves showed an increased cell killing after proton irradiation compared to X-ray irradiated cells (Figure 2a–f). This resulted in relative biologic effectiveness at 10% survival (RBE_10_) values of 1.51, 1.31, and 1.20, in MCF-7, MDA-MB-231, and MCF-10A cell lines, respectively. All observed RBE values are close to the RBE value of 1.1, which is used in clinical practice. The proton RBE was further enhanced by pre-treatment with CUDC-101, from 1.15 to 1.34 in the MCF-7 cell line and from 1.2 to 1.34 in MCF-10A cell line (Table 2). In the case of the MDA-MB-231 cell line, combination treatment of 2 Gy protons and CUDC-101 and 2 Gy X-rays and CUDC-101 yielded similar values, as shown in Figure 2d, which inferred that the differential effect of two radiation types might be obscured by the effect of CUDC-101. Accordingly, the sensitization enhancement ratio (SER) after X-ray irradiation was higher (2.09) than the proton SER (1.77), which suggested that in the MDA-MB-231 cell line, an increased biologic effect can be anticipated after CUDC-101 and X-rays compared to combination therapy of protons and CUDC-101. The MDA-MB-231 cell line also exhibited a higher RBE compared to the other two cell lines, suggesting increased sensitivity to proton irradiation. The SER values observed for the MCF-7 and MCF-10A cell lines were higher for protons compared to X-rays, which indicates increased sensitisation after proton irradiation compared to X-ray irradiation. The proton SER for MCF-7 was higher (1.50) than that determined in MCF-10A cell line (1.23), and the X-ray SER values of the cell lines were comparable (1.16 and 1.10 in MCF-7 and MCF-10A cell lines, respectively).

### 2.3. Effect of CUDC-101 on Radiation-Induced DNA DSB Formation and Repair

**Figure 3:**
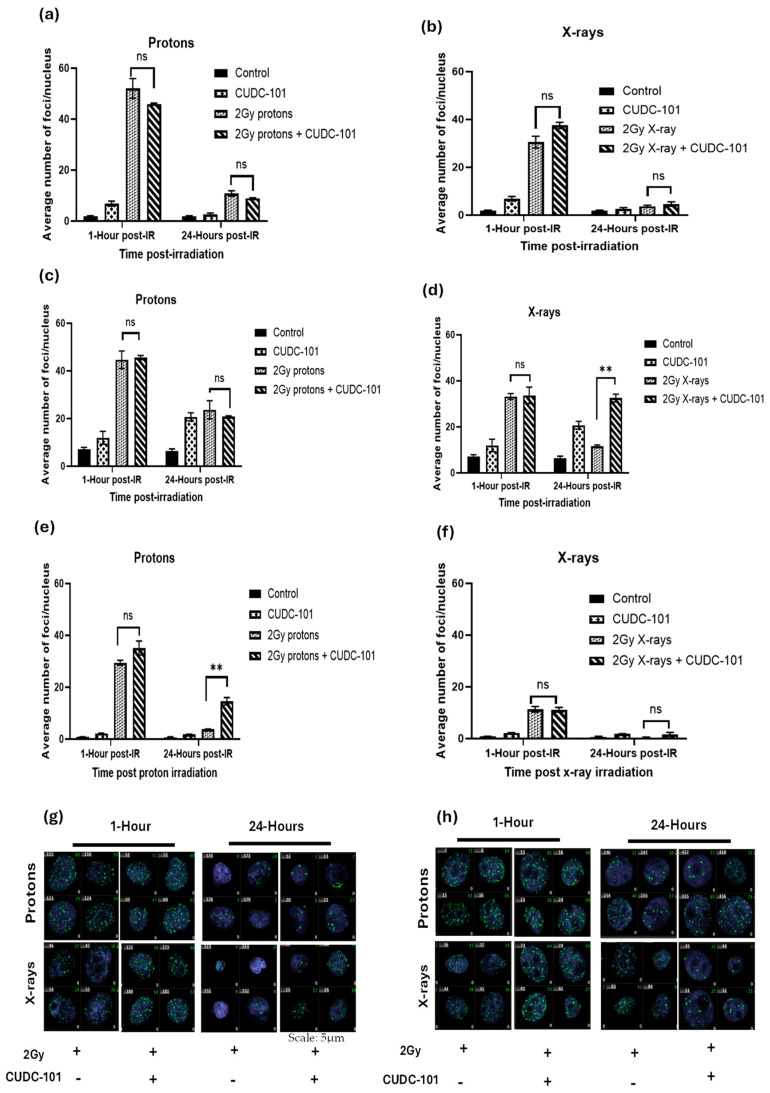
Effect of CUDC-101 combined with protons or X-rays in MCF-7 (**a**,**b**), MDA-MB-231 (**c**,**d**), and MCF-10A (**e**,**f**) cell lines. Histograms show the mean ± SD of three independent experiments (n = 3) ** *p* = 0.0037 in MDA-MB-231 and ** *p* = 0.0088 in MCF-10A cell lines. Representative images of γ-H2AX foci at 1 h and 24 h post-irradiation in MCF-7 (**g**) and MDA-MB-231 cell lines (**h**). Images are obtained at 40× magnification using a Metafer 4 scanning system. Analysis was performed using unpaired two-tailed Student’s *t* test, ** *p* (<0.002).

To assess DNA damage induction and repair after combination treatment with CUDC-101 and irradiation, γ-H2AX foci assays were performed as molecular markers of DNA double strand break (DSB) and repair. Cells were pre-treated with IC_50_ concentrations of HDACi for 24 h and irradiated with protons or X-rays. γ-H2AX foci assays were performed at 1 h and 24 h post-irradiation with 2 Gy 148 MeV mid-spread-out Bragg peak (SOBP) protons or 250 kVp X-rays. Overall, an increased number of γ-H2AX foci were noted post-irradiation with protons compared to X-irradiation in all three cell lines (Figure 3a–f). Further, in comparison to X-ray irradiated cells, an increased number of persisting γ-H2AX foci at 24 h post-proton irradiated cells was observed, which suggested that the type of damage induced by protons is complex in nature and more difficult to repair (Figure 3a–f).

In the MCF-7 cell line, at 24 h post irradiation, a significant reduction in the number of γ-H2AX foci was noted after irradiation protons (*p* < 0.0047) or X-rays (*p* < 0.0009) as well as in CUDC-101 pre-treated cells (*p* < 0.0001), which suggests that addition of the CUDC-101 did not impair the repair of the DNA DSB (Figure 3a,b). Comparison of the combination treatment (CUDC-101 and 2Gy) and irradiation alone (2 Gy protons), resulted in a non-significant result with *p* values of 0.1580 and 0.1319 at 1 h and 24 h post irradiation, respectively. Comparison of combination treatment of 2 Gy and X-irradiation and X-rays also yielded a non-significant result with *p* values of 0.0718 and 0.3018 at 1 h and 24 h post irradiation, respectively. Although not statistically significant, it was noted that proton irradiation alone yielded a higher number of γ-H2AX foci as compared to combination treatment of proton and CUDC-101 at 1 h post irradiation in this cell line (Figure 3a). This was not observed after irradiation with X-rays (Figure 3b).

In the MDA-MB-231 cell line, the number of γ-H2AX foci induced by radiation alone (protons or X-rays) and those induced by combination of radiation and CUDC-101 were not statistically significant (*p* = 0.7672) at 1 h post irradiation (Figure 3c,d). A significantly reduced (*p* = 0.0003) but notable number of retained γ-H2AX foci was observed after combination therapy with proton and CUDC-101 as well as proton irradiation alone (Figure 3c). For X-ray irradiation, the number of retained γ-H2AX foci at 24 h after combined treatment remained high and the decrease in the number of γ-H2AX foci was not statistically significant (*p* = 0.4262) compared to the 1 h time point (Figure 3d). Taken together, the findings suggest repair impairment in the MDA-MB-231 cell line and a high sensitivity to CUDC-101, which is also observable in the unirradiated CUDC-101 control samples. This is reflected as an increase in the number of γ-H2AX foci induced by CUDC-101 monotherapy at 24 h as compared to 1 h (Figure 3c,d).

In the MCF-10A cell line, the only non-malignant cell line included in the study, an overall reduced number of γ-H2AX foci were noticed compared to the other two cell lines. A statistically significant (*p* = 0.008) higher number of remaining γ-H2AX foci at 24 h was noted with 2 Gy protons and CUDC-101 compared to proton irradiation alone (Figure 3e). Almost complete repair was noted after proton and X-ray irradiation alone and in the combination treatment of X-rays and CUDC-101 at 24 h (Figure 3f). Representative immunohistochemistry images of MCF-7 and MDA-MB-231 cell lines are shown in Figure 3g and Figure 3h, respectively.

### 2.4. Impact of CUDC-101 and Radiation on Apoptosis in Breast Cell Lines

**Figure 4:**
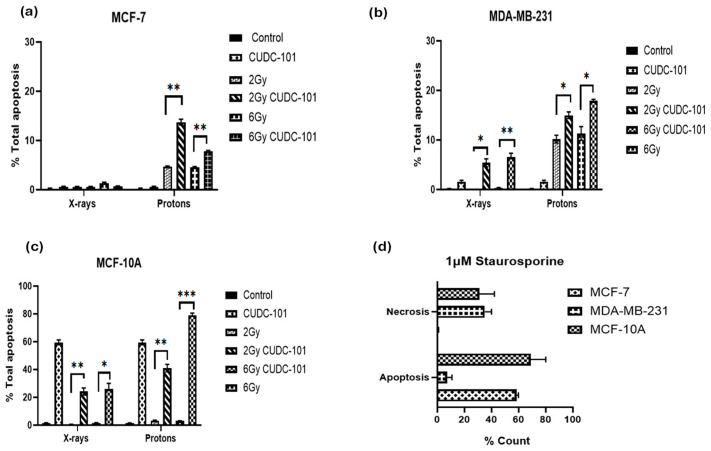
Induction of apoptosis and necrosis at 48 h post treatment with CUDC-101 combined with protons or X-rays in MCF-7 (**a**), MDA-MB-231 (**b**), and MCF-10A (**c**) cell lines. Induction of apoptosis and necrosis at 24 h after treatment with 1 µM staurosporine in the three cell lines (**d**). Histograms show the mean ± SD of three independent experiments (n = 3). Comparisons were conducted using unpaired two-tailed Student’s *t* test, *** *p* (<0.0003) ** *p* (<0.002), * *p* (<0.05).

To investigate the induction of apoptosis after treatment with CUDC-101, radiation (protons or X-rays), or combination therapy of CUDC-101 and radiation, the Annexin V/PI apoptosis assay was performed. Apoptosis and necrosis were assessed at 48 h post-irradiation with 2 Gy and 6 Gy protons or X-rays, as well as after combination of CUDC-101 and radiation (proton or X-rays). In all three cell lines, increased apoptosis levels were observed post proton-irradiations as compared to X-ray irradiations (Figure 4a–c). Pre-treatment with CUDC-101 significantly increased the level of proton-induced apoptosis after 2 Gy (*p* = 0.0020) and after 6 Gy (*p* = 0.0011) in MCF-7 cell line (Figure 4a). Similarly, in the MDA-MB-231 cell line, a significant increase was observed in CUDC-101 pre-treated samples after 2 Gy (*p* = 0.0219) and 6 Gy (*p* = 0.0216) (Figure 4b). In the spontaneously immortalised MCF-10A cell samples, increased apoptotic fractions were observed in the CUDC-101 treated MCF-10A cells (Figure 4c). Further, treatment with 1 µM of apoptosis inducer staurosporine induced apoptosis in MCF-7 and MCF-10A cell lines, whereas necrosis was induced in the MDA-MB-231 cell line at 24 h after treatment (Figure 4d).

**Figure 5:**
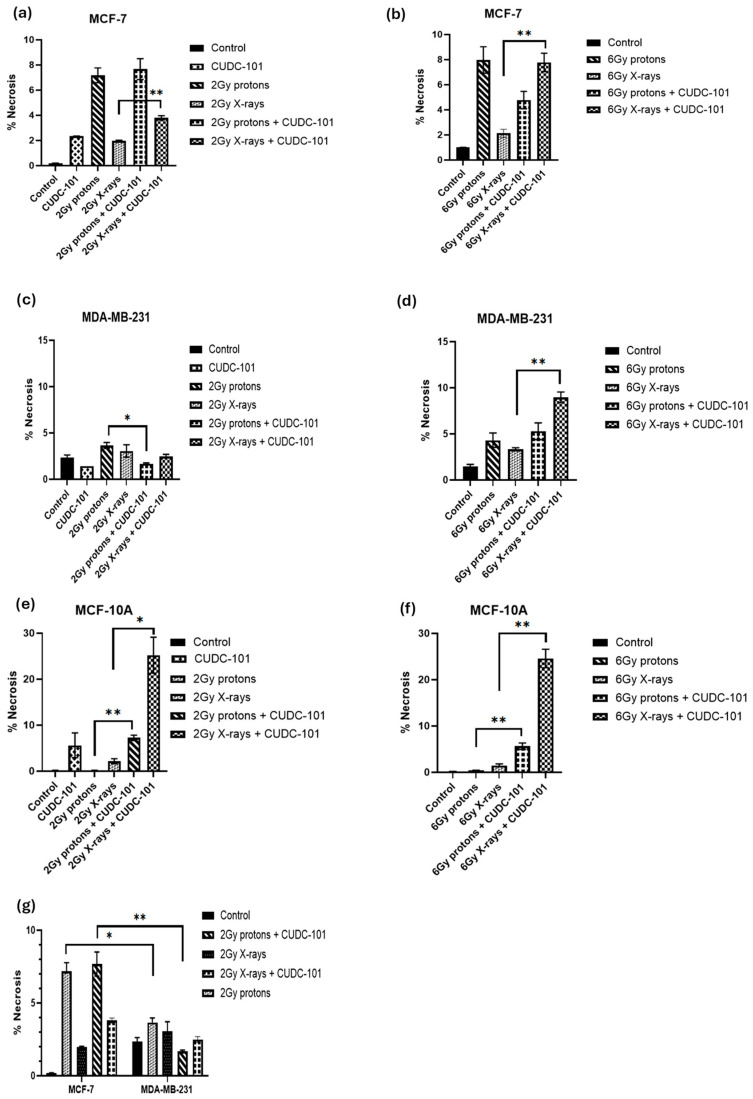
Induction of necrosis at 48 h post treatment with CUDC-101 combined with protons or X-rays in MCF-7 (**a**,**b**), MDA-MB-231 (**c**,**d**), and MCF-10A (**e**,**f**) cell lines. Increased amounts of necrosis after treatment with 2 Gy protons and 2 Gy protons and CUDC-101 in MCF-7 cell line compared to MDA-MB-231 cell line (**g**). Histograms show the mean ± SD of three independent experiments (n = 3). Comparisons were conducted using unpaired two-tailed Student’s *t* test, ** *p* (<0.002), * *p* (<0.05).

In addition, in the MCF-7 cell line, increased necrosis was noted after proton irradiations compared to X-ray irradiations (*p* = 0.0063). Pre-treatment with CUDC-101 further increased levels of necrosis after X-ray irradiation in this cell line (*p* = 0.0051) (Figure 5a,b). A different observation was made in the MDA-MB-231 cell line, pre-treatment with CUDC-101 reduced levels of necrosis after proton irradiation (*p* = 0.0136) and X-rays (*p* = 0.3647). Comparison of levels of necrosis between the two cell lines showed increased amounts of necrotic cell populations in the MCF-7 cell line compared to the MDA-MB-231 cell line after protons (*p* = 0.0174) and protons and CUDC-101 (*p* = 0.0095) (Figure 5g). Representative images of apoptosis profiles are included in Supplementary Material (Figure S1).

### 2.5. Effect of CUDC-101 and Radiation on Cell Cycle Progression

**Figure 6:**
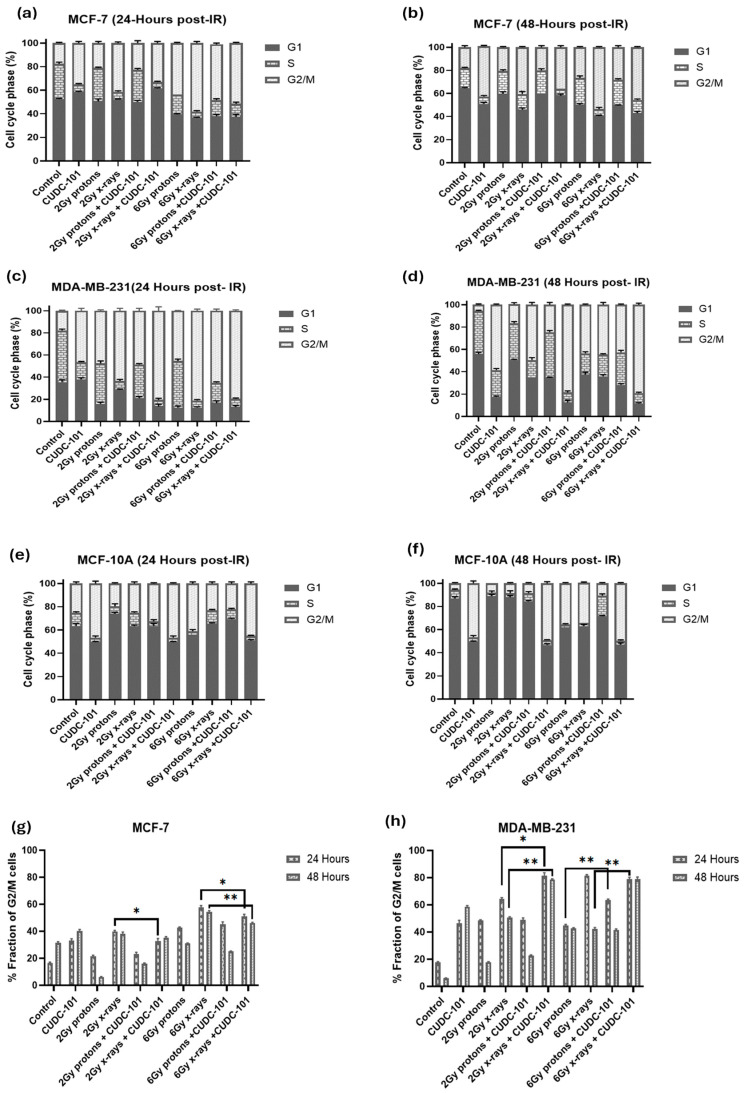
Quantification of the effect of CUDC-101 alone and in combination with X-rays and protons on cell cycle progression in MCF-7 (**a**,**b**,**g**) MDA-MB-231 (**c**,**d**,**h**); and MCF-10A (**e**,**f**) cell lines. Data represent the mean ± SD of three independent experiments (n = 3). Comparisons were conducted using two-tailed Student’s *t* test, * *p* < 0.05, ** *p* < 0.008.

Cell cycle progression after treatment with CUDC-101 and radiation was assessed using propidium iodide with RNase staining. For all cell lines, an increased fraction of cells was observed in the G2/M phase of the cell cycle at 24 h post-irradiation with 6 Gy protons or X-rays, indicating a G2/M cell cycle arrest (Figure 6a–f). Also, in all three cell lines, mono-treatment with CUDC-101 induced G2/M cell cycle arrest at 24 h, which persisted at 48 h (Figure 6a–f). In the MCF-7 and MDA-MB-231 cell lines, an increase in fraction of G2/M cells was also observed at 48 h in X-ray irradiated cells compared to proton-irradiated cells at doses of 2 Gy (*p* = 0.0012) and at 6 Gy (*p* = 0.0007). This increase was more evident in the MDA-MB-231 cell line compared to the MCF-7 cell line (Figure 6g,h). Furthermore, compared to radiation treatment alone, pre-treatment with CUDC-101 had a minimal effect on the cell cycle progression in MCF-7 cells at neither 24 h (*p* = 0.4929 for 2 Gy and *p* = 0.0532 for 6 Gy) nor 48 h (*p* = 0.6985 for 2 Gy and *p* = 0.3118 for 6 Gy) post proton-irradiation as evidenced by comparable G2/M fractions at these timepoints (Figure 5g). However, in the MDA-MB-231 cell line, pre-treatment with CUDC-101 increased the G2/M fraction after exposure to both 2 Gy *p* = 0.0030) and 6 Gy (*p* = 0.0027) X-rays which was maintained at 48 h post irradiation (Figure 6h). It seems sensible to associate the increased fraction of G2/M cells after X-ray irradiations to the reduced levels of apoptosis and necrosis that were seen in the MCF-7 and MDA-MB-231 cell lines at 48 h post irradiation. In this instance, the increased G2/M could be an indicator of mitotic catastrophe as a mode of cell death after x-irradiations. Similarly, in the MCF-10A cells, increased G2/M fractions were noted in CUDC-101 pre-treated compared to radiation alone. Cell cycle profiles are included in the Supplementary Material (Figure S2).

## 3. Discussion

### 3.1. CUDC-101 Increases Sensitivity of MCF-7, MDA-MB-231 and MCF-10A Cell Lines to Proton and X-Ray Irradiation

Our data show that the multi-target inhibitor CUDC-101 enhances the response to both protons and X-rays in MCF-7 and MCF-10A breast cell lines. The enhancement was most notable after proton irradiation, with proton SER values of 1.50 and 1.23 and X-ray SER values of 1.16 and 1.10 in MCF-7 and MCF-10A cell lines, respectively. Similarly, in hepatocellular carcinoma (HCC) cell lines, Choi et al. reported higher proton SER values of 1.25 and 1.21 compared to X-ray SER values of 1.15 and 1.11, using Panobinostat in Huh7 and Hep3B cells, respectively. Yu et al. also reported Valproic acid (VPA)-mediated RBE_10_ value of 1.17 compared to RBE_10_ value of 1.08 without VPA in Hep3B cells after treatment with 6 MV photons [31]. In another study, Gerelchuluun et al. reported an RBE_10_ value of 1.24 and proton SER values of 1.31 and 1.16 compared to γ-ray SER values of 1.43 and 1.08 in lung carcinoma (A549) and normal fibroblast (AG1522) cell lines, respectively, following treatment with 2 µM of SAHA [29]. Although different HDACi and different cell lines were used in the mentioned studies, the findings are consistent with those of the current study, where higher RBE and SER values were reported for proton compared to X-rays. The enhanced radiation response in the MCF-7 cell line indicate a potential benefit of using CUDC-101 and protons in treatment of breast tumours with oestrogen, progesterone, and her-2 positive molecular sub-types. Previous studies have asserted that HDACs are not overexpressed in normal tissues, which leads to minimal effect of HDACi on normal tissues [17,41,42]. While our study indicates SER of 1.10 and 1.23 in the normal breast cell line (MCF-10A) following pre-treatment with CUDC-101, these values were lower than the ones observed in the malignant cell lines. It should be noted that the former studies used SAHA, which inhibits HDACs only, whereas in the current study CUDC-101, which inhibits HDACs, EGFR and HER-2, was used. Therefore, the effect of CUDC-101 observed in the normal cell line can be attributed to inhibition of EGFR, which is important for growth and maintenance of MCF-10A cells [43].

A different effect was seen in the MDA-MB-231 cell line in the current study, with a higher X-ray SER value of 2.09 compared to proton SER value of 1.77, which implies that in this cell line, CUDC-101 is best combined with X-rays for maximal therapeutic benefit of the combination treatment. It is yet to be determined if a similar result will be seen using other triple-negative breast cell lines. In addition, the RBE_10_ value of 1.31 for the MDA-MB-231 cell line indicates that protons are more effective than X-rays to inhibit the proliferation capacity, at a higher rate than the RBE_10_ values observed in the MCF-7 and MCF-10A cell lines. Previous studies have associated higher RBE values to DNA damage repair capacity of the cell line. In other words, cell lines with higher RBE values, were reported to be deficient in DNA repair capacity [44,45].

### 3.2. Proton Irradiation Induces Increased DNA Damage That Is Not Easily Repaired in Breast Cell Lines

The complexity of DNA damage induction and repair after proton therapy remains a subject of discussion [46]. Contrary to X-rays that have no mass and no charge, protons are charged particles with a larger mass, which can create more direct and complex DNA damage. Previous studies reported an increased number of γ-H2AX foci that are larger in size after proton irradiation as compared to X-rays in different cell lines [47,48,49,50,51,52,53]. Other studies reported that SOBP protons induced increased γ-H2AX foci, which were larger in size and reached maximum point at 1 h post irradiation, whereas maximal γ-H2AX foci count was reached at 30 min post irradiation with X-rays and plateau protons, which were also smaller in size and were resolved at 6 h post irradiation [47,50,54]. Gerelchuulun et al. reported a 1.2–1.6-fold increase in γ-H2AX foci in ONS76 medulloblastoma and MOLT4 leukaemia cells after proton irradiation compared to 10 MV X-rays [51]. In another study, irradiation with SOBP protons induced more clustered DNA damage, whereas entrance plateau protons induced mixed-type damage that consisted of clustered and non-clustered DNA damage [50]. Consistent with these studies, a significantly increased number (1.4–1.5-fold) of γ-H2AX foci was observed at 1 h post-irradiation with 2 Gy SOBP protons compared to 2 Gy X-rays in all three cell lines (Figure 3a–h). The fact that persisting γ-H2AX foci at 24 h post irradiation were observed mainly after proton irradiation (Figure 3b,c), suggests that the type of DNA damage induced by protons is difficult to repair, supporting the assertions of complex DNA damage [47,50,51,53,55,56,57].

Of relevance is also the ongoing discussions about differential requirement of DNA DSB repair pathways following protons and X-rays [47,55]. Previous studies reported that post proton irradiation, the error free homologous repair (HR) is preferred, and non-homologous end joining (NHEJ) is preferred after irradiation with X-rays [53,55]. Latter reports indicated that HR is required post proton irradiation due to the complex nature of the DNA damage, but NHEJ is also indispensable for repair of proton-induced DSB [45,47,57]. Further evidence pointed out that irrespective of the type of radiation, the initial fast repair is conducted by NHEJ, and HR occurs at a later stage [53,58,59]. In a recent report by Lohberger et al., mismatch repair (MMR) and nucleotide excision repair (NER) repair pathways together with HR and NHEJ pathways were found to be activated mainly post-proton irradiation in chondrosarcoma cells [52]. These studies bear relevance to the observed prolonged appearance of γ-H2AX foci after proton irradiation as compared to x-irradiated cells, particularly in the MDA-MB-231 cell line in the current study. Consistent with the notion that cell lines with higher RBE values have defective repair pathways, Lee et al. reported that MDA-MB-231 cells are deficient in HR, base excision, and nucleotide excision repair (NER) [60]. This would explain the increased retention of γ-H2AX foci in MDA-MB-231 cell line and increased sensitivity to proton irradiation (RBE_10_ of 1.31) compared to the MCF-7 cell line (RBE_10_ of 1.15). As previously mentioned, the HDACi-mediated RBE was however lower than the proton RBE due to the compounding effect of DNA damage that is induced CUDC-101 in the MDA-MB-231 cell line, which was not seen in the other cell lines. Several studies also reported having observed increased sensitivity to proton irradiation in cells that are deficient in HR machinery [47,53,55,56,61].

Limited studies have been conducted on combination therapy of HDACi and proton irradiation with respect to DNA DSB induction and repair. A 3 h pre-treatment with 1 mM HDACi Valproic acid (VPA) and mid-SOBP protons prolonged appearance of γ-H2AX foci in Hep3B and Huh7 hepatocellular carcinoma cell lines [31]. Pre-treatment with 5 nM Panobinostat increased the γ-H2AX foci yield at 24 h post irradiation with 6 Gy mid-SOBP protons in Huh7 and Hep3B hepatocellular carcinoma cell lines [28]. In NFF28 normal fibroblast cells, Johnson et al. reported resolution of γ-H2AX foci to near background levels at 24 h post-treatment with 10 µM SAHA and irradiation with 200 MeV protons [30]. The results of these earlier studies are consistent with the observation in the current study, since the retention of γ-H2AX foci was in general higher after proton irradiation in malignant cell lines compared the MCF-10A cell line. It is also worth noting that in the study by Johnson et al., 200 MeV entrance plateau protons were used, whereas 148 MeV mid-SOBP protons were used in the current study. Persisting γ-H2AX foci were detected at 24 h post treatment with 2 Gy protons and CUDC-101 in the MCF-10A cell line, which could be an indication of increased normal tissue effect.

Treatment with CUDC-101 alone resulted in an induction of γ-H2AX foci at 1 h, which increased at 24 h post treatment in MDA-MB-231 cell line (Figure 3c–e), but not in the MCF-7 and MCF-10A cell lines. Similarly, in the MDA-MB-231 cell line, an increased G2/M phase fraction in comparison to the untreated control was seen at 24 h (*p* = 0.003) and at 48 h (*p* = 0.0002). The increase in the number of γ-H2AX foci at 24 h suggests that additional γ-H2AX foci might have been induced by cell death mechanisms. Induction of DNA damage by sole treatment with HDACi has previously been reported mainly in leukaemia cells [62,63], which would explain the success of HDACi monotherapies in treating haematological malignancies with poor performance in solid tumours. In a study by Choi et al., Panobinostat alone did not induce γ-H2AX foci in hepatocellular carcinoma cell lines [28]. Further investigation is required to confirm the observations of CUDC-101-induced DNA DSB formation in the current study.

### 3.3. CUDC-101 Enhances Protons-Induced Apoptosis

The type of cell death after irradiation is mainly determined by the cell type and type of radiation. Apoptosis was formerly reported to be the main mode of cell death in haematological cancer cells whereas mitotic catastrophe was reported to be the main mode of cell death in solid tumours after irradiation [64]. Further, several studies asserted that apoptosis would be the main mode of cell death in solid tumours, through either the intrinsic or extrinsic apoptotic pathways, whereas the main mode of cell death post X-ray irradiation would be mitotic catastrophe [51,64,65,66,67,68,69,70]. Consistent with these assertions made in these reports, increased apoptosis was observed post proton irradiations, whereas minimal levels of apoptosis were noted after X-ray irradiation in all three cell lines (Figure 4a–c). Apoptosis was most notable in the MDA-MB-231 cell line at 2 Gy and 6 Gy proton irradiations, as well as after combination therapy of proton irradiation (2 Gy and 6 Gy) and CUDC-101. Although a marked number of unresolved DSB was noted at 24 h after treatment with CUDC-101 monotherapy in this cell line, minimal levels of apoptosis were noted, suggesting a different type of cell death. Indeed, cell cycle analysis in both MCF-7 and MDA-MB-231 cell lines showed increased G2/M arrest at 48 h after CUDC-101 monotherapies, as well as after combination treatments of CUDC-101 and X-ray irradiation (Figure 5a–d), which suggested induction of mitotic catastrophe. Similar observations were made by Schlaff et al. who reported induction of mitotic catastrophe in glioblastoma cell line after CUDC-101 treatment [14]. Keeping with the argument that CUDC-101 and X-ray monotherapies induces mitotic catastrophe, the combination therapy of X-rays and CUDC-101 was expected to result in higher levels of mitotic catastrophe (G2/M) compared to proton irradiations and CUDC-101. Certainly, Figure 5d,e show an even higher proportion of G2/M cells after combination therapy of 2 Gy X-rays and CUDC-101 compared to monotherapies with either X-rays or CUDC-101 in the MDA-MB-231 cell line.

In the MCF-7 cell line, the levels of apoptosis and necrosis were comparable at the lower dose of 2 Gy (Figure 4a,b). The levels of necrosis exceeded that of apoptosis at doses of 6 Gy, as shown in Figure 4c. Several reports have asserted that MCF-7 cells are deficient in caspase 3 and therefore lack the morphological features associated with apoptosis [71,72]. Natarajan et al. reported that MCF-7 cells can switch to a necroptosis pathway after treatment with HDACi SAHA [73]. Necroptosis is a caspase-independent mechanism of programmed cell death that is activated when apoptosis is blocked and it bears mechanistic similarity to apoptosis and morphological similarity to necrosis [74]. Treatment with 1 µM of apoptosis inducer staurosporine, induced more apoptosis in the MCF-7 cell line compared to the MDA-MB-231 cell line at 24 h post treatment (Figure 6d). In earlier studies, staurosporine, at a concentration of 1 µM, was reported to induce apoptosis in MCF-7 cells through partial activation of caspase-6 [71]. The authors also noted that apoptosis occurred earlier (16 h), with the absence of typical apoptotic morphology and absence of apoptotic bodies in the MCF-7 cell line compared to T47D cells [71]. To the contrary, Poliseno et al. reported that at 5 h post treatment with staurisporine at 1 µM induced necrosis in MCF-7 cell lines expressing low anti-apoptotic Bcl-2 protein [75]. Taken together, these studies imply that necrosis is induced earlier, and apoptosis is only detectable at a later stage in MCF-7 cells. In view of the mentioned overlapping similarities between necroptosis, necrosis, and partial apoptosis in this cell line, it seems reasonable to assume that what was reported in previous literature, as well as in the current study, might have been necroptosis. In view of the fact that very little apoptosis was observed in all three cell lines, it is advisable that other modes of cell death such as mitotic catastrophe, autophagy, and necroptosis be investigated in follow-up in vitro work, particularly in the MCF-7 cell line, which is caspase 3-deficient [76].

The increased apoptosis seen in CUDC-101-treated cells in the MCF-10A cell line is thought to be due to inhibition of EGFR (Figure 4c). Increased apoptosis was seen mainly after treatment with protons and CUDC-101 compared to X-ray irradiated cells (Figure 4c). Further, lower levels of apoptosis were seen after treatment with 2 Gy X-rays and CUDC-101 compared to 6 Gy X-rays and CUDC-101. These findings imply that, if CUDC-101 is considered for use in triple-negative breast cancer, it might be better to combine it with X-rays to reduce normal tissue reactions. Notwithstanding, in a Phase 1 study of 275 mg/m^2^ CUDC-101 in combination with cisplatin and X-ray radiation in squamous cell head and neck cancers, out of the 12 patients that enrolled for the study, 5 patients discontinued CUDC-101 due to adverse side effects. CUDC-101 was administered three times a week for one week before starting with radiation and cisplatin and was concurrently administered with cisplatin and radiation in a fractionated regime up to a total dose of 70 Gy. The authors suggested alternate scheduling of CUDC-101 and using different routes of administration to minimize adverse effects [76]. Subsequently, in another Phase I trial in advanced solid tumours, Schlaff et al. reported that intravenous administration of CUDC-101 for 1 h for 5 consecutive days every 2 weeks were well tolerated. The authors recommended a dose of 275 mg/m^2^ to be used [77]. A Phase I study (NCT01702285) to assess safety and tolerability of orally administered CUDC-101 was terminated for unknown reasons.

### 3.4. CUDC-101 Induces G2/M Cell Cycle Arrest and Enhances X-Irradiation-Induced G2/M Cell Cycle Arrest

**Figure 7:**
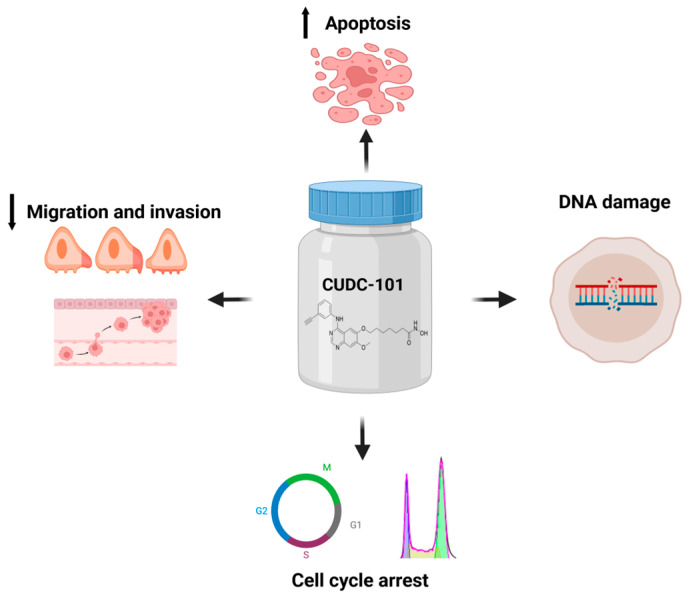
Summary illustration of the cellular effects of CUDC-101.

Previous studies highlighted the important role that HDACs play in cell cycle progression. In particular, HDAC 3 and HDAC 10 have been implicated in mediating progression through the G2/M phase of the cell cycle [78,79,80]. HDACs 2, 3, 5, and SIRT2 have also been implicated in fascilitating exit from mitosis stage [78,81,82,83]. HDACi, therefore, induces CDK-inhibitor p21, to induce cell cycle arrest [78]. Monotherapy with 5 nM of HDACi panobinostat induced G2/M arrest from 24.3% to 51.4% at 24 h post-treatment in Huh7 HCC cell lines. Pre-treatment with 5 nM panobinostat also increased G2/M proportions of cells after 6 Gy X-ray or proton irradiation [28]. The amounts of G2/M cells seen after single panobinostat treatment and after combination therapy of panobinostat and proton- or X-ray irradiation were not statistically signinficant [28]. In another study, 6 Gy photons or 6 Gy protons increased proportions of G2/M cell to 71% and 70%, respectively in Hep3 HCC cell line [31]. Pre-treatment with 1 mM of HDACi VPA before radiation further increased G2/M fraction from 73.4% to 80.1% at 24 h and from 59.9% to 58.6% at 72 h [31]. In the current study, similar to the mentioned studies, 6 Gy protons and 6 Gy X-rays increased G2/M cells at 24 h compared to the untreated control. Also, similar to Choi et al., treatment with CUDC-101 monotherapy increased G2/M cell cycle arrest in all three breast cell lines at 24 h. The increase was most notable in the MDA-MB-231 and MCF-10A cell lines, indicating increased toxicity of CUDC-101 in these cell lines. Contrary to the mentioned studies, increased G2/M was seen in X-ray-irradiated cells compared to the proton-irradiated cells, with or without HDACi pre-treatment in the current study. As previously mentioned, this differential increase in G2/M fraction after X-rays suggested mitotic catastrophe as a mode of cell death. The summary of the cellular effects of CUDC-101 is presneted in Figure 7.

## 4. Materials and Methods

### 4.1. Cell Cultures

MCF-7 and MCF-10A (gifted by the Physiology Department, University of Pretoria) cells were cultured in Dulbecco’s Modified Eagle’s Medium F-12 (DMEM-F12; Gibco^TM^, Thermo Fisher Scientific, Sandton, South Africa) and Ham’s F-12 (Gibco^TM^, Thermo Fisher Scientific, Sandton, South Africa) supplemented with 10% foetal bovine serum (FBS) (Gibco^TM^, Thermo Fisher Scientific, Sandton, South Africa), 100 μg/mL penicillin (Gibco^TM^, Thermo Fisher Scientific, Sandton, South Africa), and 100 μg/mL streptomycin for bacterial contamination. MCF-10A medium was further supplemented with epidermal growth factor (EGF) (20 ng/mL final concentration) (Gibco^TM^, Thermo Fisher Scientific, Sandton, South Africa) and hydrocortisone (0.5 mg/mL final concentration) (Sigma-Aldrich, St. Louis, MO, USA).

MDA-MB-231 cells (gifted by the Department of Natural Sciences, University of Western Cape) were cultured in Roswell Park Memorial Institute (RPMI) 1640 (Gibco^TM^, Thermo Fisher Scientific, Sandton, South Africa) supplemented with 10% FBS, 100 μg/L penicillin and 100 μg/mL streptomycin (Sigma-Aldrich, St. Louis, MO, USA).

All cell lines were cultured in T275 or T75 cell culture flasks (Thermo Fisher Scientific, Sandton, South Africa) under standard conditions in a humidified incubator at 37 °C, 5% CO_2_ (Forma series 3 water jacketed incubator, Thermo Fisher Scientific, Waltham, MA, USA). Cell growth was assessed over 24 h intervals and sub-cultured once 80% confluence was reached.

### 4.2. Histone Deacetylase Inhibitor

**Figure 8:**
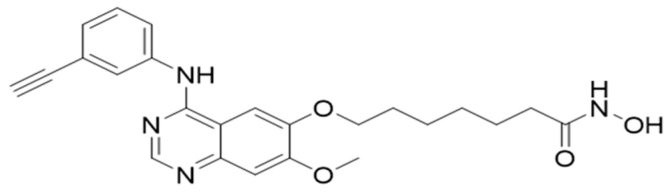
Molecular structure of CUDC-101.

CUDC-101 (molecular weight of 434.49) Figure 8, was purchased from Sigma Aldrich (Sigma-Aldrich, St. Louis, MO, USA) and 1 mM stock solution was prepared according to the manufacturer’s instructions (5 mg of CUDC-101 was resolved in 11.5077 mL dimethylsulfide (DMSO) (Biotechnology Hub, Johannesburg, South Africa) and stored at −20° for short term storage and at −80° for long term storage.

### 4.3. Irradiations

Photon irradiations were performed using the 250 kVp X-Rad 320 unit (Precision X-ray, Madison, WI, USA) at a mean dose rate of 0.69 Gy/min at a Source Surface Distance (SSD) of 50 cm. Calibrations of the unit were performed according to the Technical Report Series-398 (TRS-398) protocol, with a Farmer 117 chamber for which a chamber calibration factor has been obtained from the National Metrology Institute of South Africa (NMISA).

Proton irradiations were performed at the Trento Institute for Fundamental Physics and Application (TIFPA). An SOBP beam of 2.5 cm has been produced, as detailed in Tommasino et al. through a 2D rang modulator applied to a beam with initial energy of 148 MeV/u and enlarged with a dual ring system to a lateral profile maintaining a 98% dose uniformity across a 6 cm diameter. The beam was calibrated with EBT gafchromic film and Markus chamber measurements. The cells were exposed after 11 cm of solid water slabs, corresponding to 11.45 cm of water [84]. For both X-ray and proton irradiations, cells were irradiated in 5 mL media in T25 flasks.

### 4.4. Cell Proliferation Assays

Cell proliferation assays were conducted using the thiazolyl blue tetrazoliumbromide (MTT) cell viability assay kit. Cells were seeded at a pre-determined density of 3000 cells/well in 96-well plates and allowed to attach. Cells were treated with different concentrations of the HDACi (SAHA or CUDC-101) ranging from 0 µM to 20 µM and incubated for a further 72 h. A 20 μL of a 5 mg/mL stock solution of MTT (Sigma-Aldrich, St. Louis, MO, USA) was added to each well and incubated for a further 4 h to allow formazan formation. MTT-containing media was carefully removed and 100 μL of DMSO was added to each well. The formation of formazan in the viable cells was monitored by measuring absorbance at wavelengths of 595 nm on a spectrophotometer to determine the half-maximal inhibitory concentrations (IC50) values for HDACi SAHA and CUDC-101 for the different cell lines.

### 4.5. Colony Survival Assays (CSA) and RBE Analysis

Cells (250–1500) were seeded in 6-well plates (Whitehead Scientific, Cape Town, South Africa) and allowed to attach overnight. Cells were treated with CUDC-101 at a concentration of 0.6 µM, 2.7 µM, and 0.3 µM for MCF-7, MCF-10A, and MDA-MB-231, respectively, for 24 h and irradiated with 0, 2, 4, 6, and 8 Gy of 250 KeV X-rays or 148 MeV SOBP protons. The cells were returned for incubation for a further 8–14 days to allow for colony development. Once colonies of approximately 50 cells were formed, they were fixed with methanol and stained with 2% crystal violet dissolved in methanol and left to dry overnight. Colonies were manually counted, and the size was validated by microscopic inspection. Plating efficiency was calculated under untreated conditions using the equation:

(1) $$Plating\;efficiency\;(PE)=\frac{number\;of\;colonies\;counted}{number\;of\;cells\;seeded}\times 100\%$$

Plating efficiency was used to normalise the surviving fractions for HDACi and radiation-induced cell death. The surviving fraction of cells was calculated using the equation:

(2) $$Surviving\;Fraction=\frac{number\;of\;colonies\;formed\;after\;treatment}{number\;of\;cells\;seeded\times PE}$$

Survival curves were plotted and analysed using GraphPad Prism Software Version 10.00 for Windows (GraphPad Software, San Diego, CA, USA). The RBE of the different treatment conditions was calculated at a survival fraction of 10%:

(3) $$RBE=\frac{Dose\;of\;photon\;radiation\;inducing\;10\%\;SF\;}{Dose\;of\;proton\;radiation\;inducing\;10\%\;SF}$$

The sensitisation enhancement ratio (SER) was calculated using the equation [28,29]:

$$SER=\frac{Radiaton\;dose\;to\;inducing\;10\%\;SF\;without\;CUDC-101}{Radiation\;dose\;inducing\;10\%\;SF\;with\;CUDC-101}$$

### 4.6. Annexin V-FITC/Propidium Iodide Apoptosis and Cell Cycle Analysis Assays

Apoptosis and cell cycle progression were analysed using flow cytometry. For the apoptosis assays, at 48 h post-irradiation, the media in which the cells were incubated was retained and combined with harvested cells before centrifugation. The cell pellet was resuspended in 100 µL 1× annexin-binding buffer and stained with with 2.5 µL Annexin V FITC and 2.5 µL propidium iodide (PI) (catalogue number 13242, Invitrogen, Thermo Fisher Scientific, Sandton, South Africa) according to the manufacturer’s instructions. Cells were incubated at room temperature (25 °C) for 15 min in the dark. An additional annexin-binding buffer was added after incubation, and the samples were analysed using the BD Accuri^TM^ C6 Plus (BD Biosciences, Johannesburg, South Africa) with 15,000–20,000 cells per measurement.

For analysis of the cell cycle, cells were harvested at 24 h and 48 h post treatments, and the cell pellet was resuspended in a solution of 100 µL propidium iodide and RNase (FxCycle^TM^ PI/RNase, Invitrogen, Waltham, MA, USA). Propidium iodide stains for both DNA and RNA, therefore the ribonuclease (RNase) digests and removes RNA to ensure that only DNA content is analysed [85]. The samples were analysed using FACSort (Beckton Dickinson, San Jose, CA, USA), with 15,000–20,000 events per measurement. Fluorescence measurements were done at 495 nm and 519 nm (peak emission) at fluorescein isothiocyanate (FITC) channel for Annexin V FITC; 536 nm and 616 nm (peak emission) at FL2 or FL3 channels for propidium iodide for all flow cytometry assays.

### 4.7. Gamma-H2AX Foci Assay

Treated cells were harvested 1 h and at 24 h post irradiation and a suspension of approximately 120,000 cells/0.25 mL was centrifuged onto coated slides (X-tra adhesive slides, Leica Biosystems, Buffalo Grove, IL, USA). Three slides were prepared for each treatment condition. The slides were fixed in freshly prepared 4% paraformaldehyde (PFA) for 20 min and washed in PBS for 5 min. Cells were then permeabilised with PBS-triton X-100 solution (GibcoTM, Thermo Fisher Scientific, Sandton, South Africa) for 10 min and blocking of no-specific antibody binding by washing 3 times in 1% bovine serum albumin (BSA) solution (Roche, Sigma-Aldrich/Merck, St. Louis, MO, USA) for 10 min per wash. Cells were incubated with phospho-histone H2A.X (Ser139) Monoclonal Antibody (3F2) antibody (Invitrogen, Biocom Africa (Pty) Ltd., Centurion, South Africa) for 1 h at room temperature, followed by washing 3 times in 1% bovine serum albumin (BSA) (Roche, Sigma-Aldrich/Merck, St. Louis, MO, USA) to remove any unbound primary antibody. Cells were then incubated for a further 1 h in the dark with rabbit anti-mouse IgG (H + L) FITC Secondary Antibody, secondary antibody (Invitrogen, Thermo Fisher Scientific, Sandton, South Africa) in a humidified chamber. Nuclear counterstaining was performed with Prolong diamond anti-fade with DAPI (Thermo Fisher Scientific, Sandton, South Africa). Slides were stored at room temperature for a minimum of 24 h and scanned automatically using the MetaCyte software module of the Metafer 4 scanning system with a 40× objective. For each slide, a minimum of 1000 cells were captured, and the average number of γ-H2AX foci per scanned slide was derived from the MetaCyte software, version 4.3.

### 4.8. Statistical Analysis

Statistical analysis was performed using Graphpad Prism version 10.2. All data was expressed as the mean the mean ± SD of three independent experiments (n = 3). Statistical significance was determined using two-tailed Student’s *t*-test, and *p* < 0.05 was considered statistically significant.

## 5. Conclusions

Positive EGFR status has long been recognised as a negative prognostic factor in breast cancer, and evidence has pointed out that EGFR is overexpressed in triple-negative cancer [40]. The marked response seen in the MDA-MB-231 and MCF-10A cell lines in this study can be attributed to inhibition of EGFR by CUDC-101. The radiation-enhancing capacity of CUDC-101 was more pronounced when combined with X-rays (SER values of 2.09 and 1.77, for X-rays and protons, respectively). The current results draw attention to the potential benefit of CUDC-101 in the management of triple-negative breast cancers as monotherapy or when combined with X-ray irradiation or proton irradiation. However, the increased toxicity of CUDC-101 on normal cells (MCF-10A) particularly when combined with protons cannot be ignored. Data from a Phase I studies have showed that CUDC-101 in combination with X-rays can be tolerated [76,84]. For these reasons, it is advisable that CUDC-101 in combination with X-rays, rather than with protons, be considered. Although the results shows that CUDC-101 has potential to enhance treatment efficacy in combination treatment with radiation, future preclinical in vivo research and clinical trials are warranted to confirm these in vitro findings.

## Figures and Tables

**Table 1:** Half maximal inhibitory concentration (IC_50_) values for the different cell lines.

Cell Line	CUDC-101 (µM)
MCF-7	0.31
MDA-MB-231	0.60
MCF-10A	2.70

**Table 2:** Radiation response parameters of MCF-7, MDA-MB-231, and MCF-10A cell lines.

Cell Line	RBE_10_	HDACi-Mediated RBE	SER_10_ (Protons)	SER_10_ (X-Rays)
MCF-7	1.15 ± 0.03	1.37 ± 0.00	1.50 ± 0.01	1.16 ± 0.03
MDA-MB-231	1.31 ± 0.01	-	1.77 ± 0.03	2.09 ± 0.02
MCF-10A	1.20 ± 0.01	1.34 ± 0.02	1.23 ± 0.04	1.10 ± 0.01

Data represent the mean ± SD. RBE: relative biologic effectiveness; SER: the sensitisation enhancement ratio.

## Data Availability

The original contributions presented in the study are included in the article/Supplementary Materials, further inquiries can be directed to the corresponding author.

## Supplementary Materials

The following supporting information can be downloaded at: https://www.mdpi.com/article/10.3390/ph17111467/s1, Figure S1: Representative images for apoptosis profiles; Figure S2: Representative images of the cell cycle profile after different treatment conditions.
