# Supplementary material for: Multi-Target Inhibitor CUDC-101 Impairs DNA Damage Repair and Enhances Radiation Response in Triple-Negative Breast Cell Line
Source: Pharmaceuticals (Basel). 2024 Nov 1;17(11):1467. doi: 10.3390/ph17111467 (PMC11597529; doi:10.3390/ph17111467)
Supplement: Supplementary file 1 [file pharmaceuticals-17-01467-s001.zip › pharmaceuticals-3268134-supplementary.pdf]

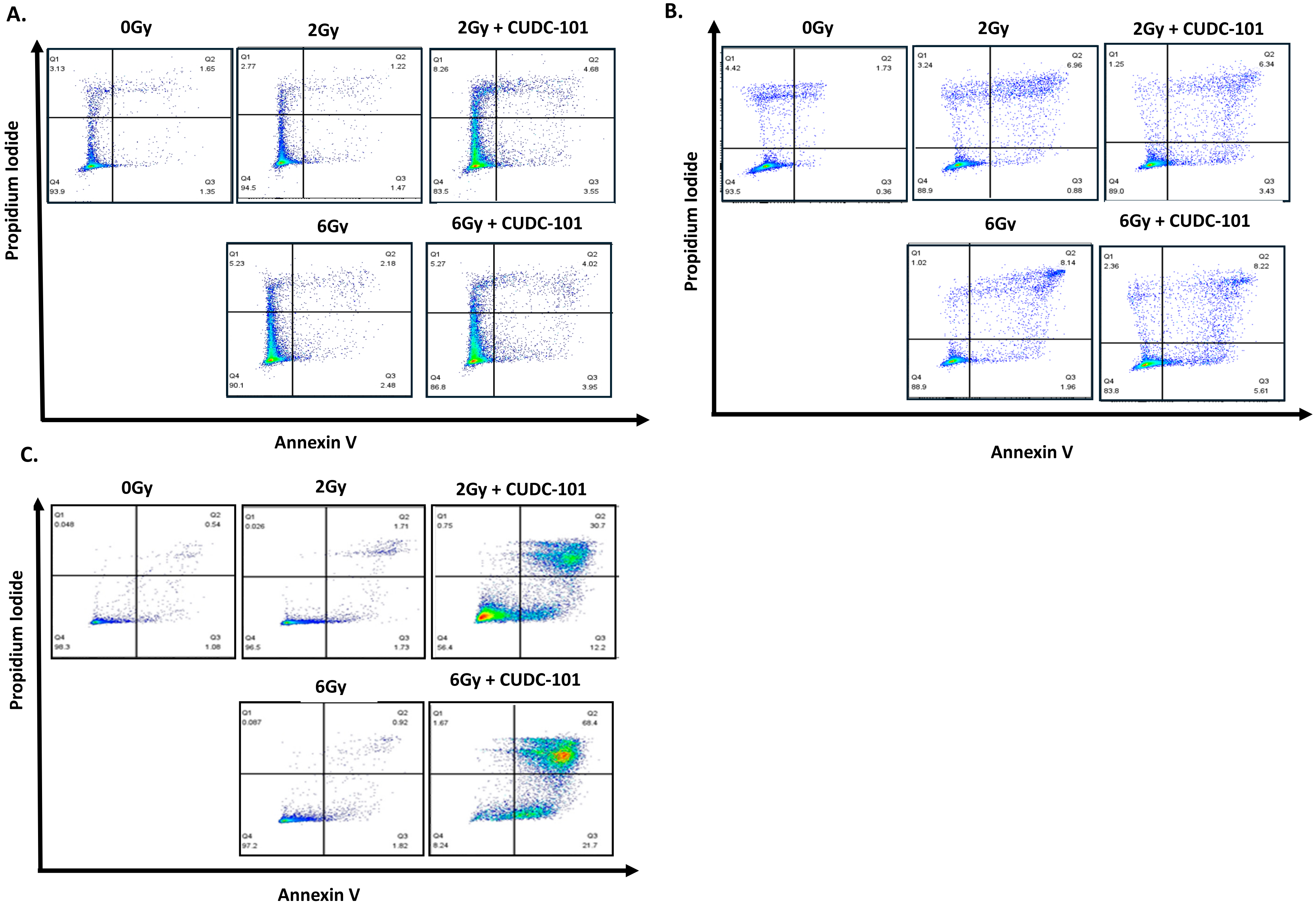

Figure S1: Apoptosis profiles of MCF-7 (A), MDA-MB-231 (B) and MCF-10A (C) cell lines post treatment with 2Gy protons, 2Gy protons and CUDC-101,6Gy protons and 6Gy protons and CUDC-101.

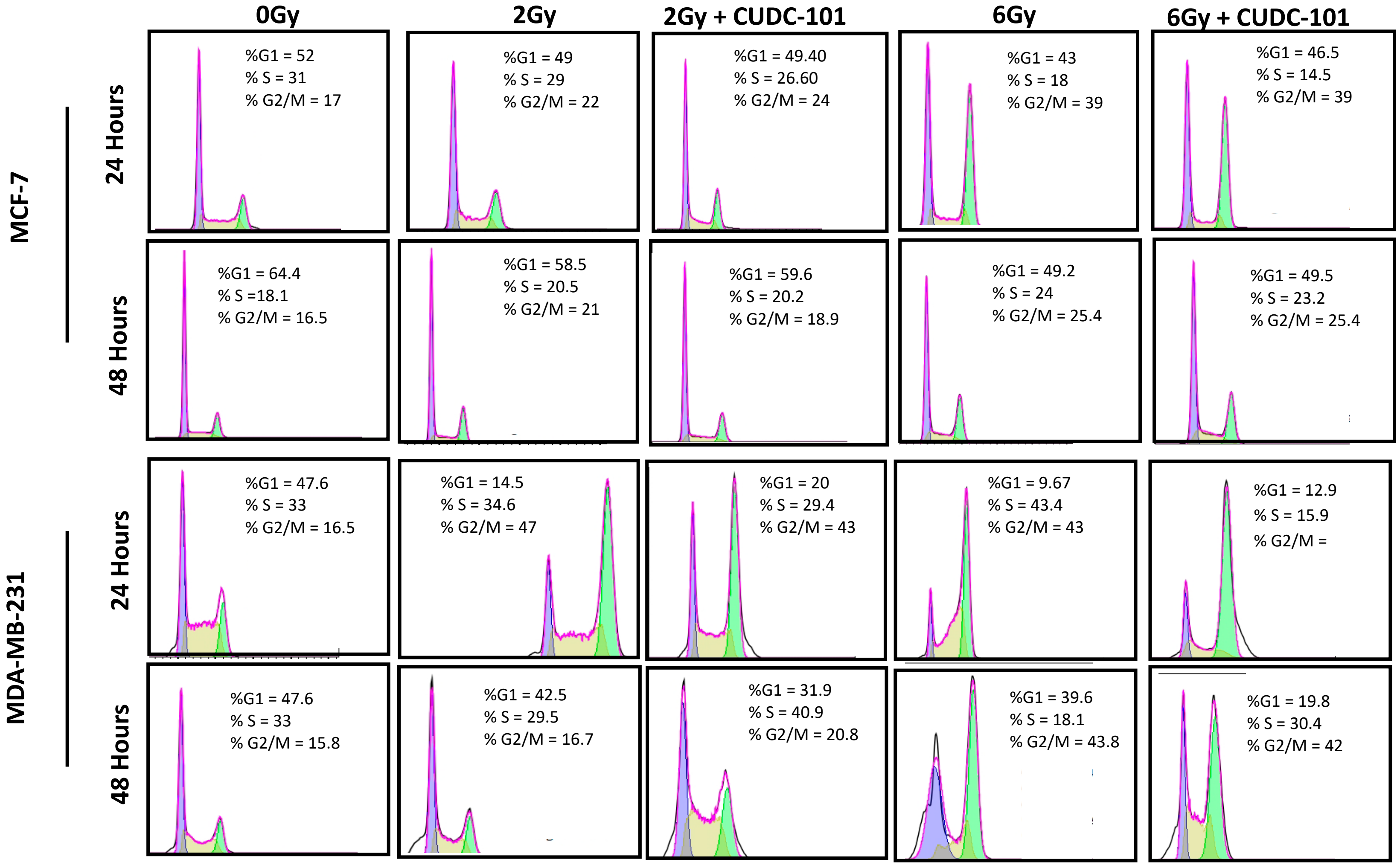

Figure S2: Cell cycle profiles of MCF-7 and MDA-MB-231 cell lines post treatment with 2Gy protons, 2Gy protons and CUDC-101,6Gy protons and 6Gy protons and CUDC-101.
